# Beyond the Intestinal Mucosa in Long-Standing Inflammatory Bowel Disease: Consequences of Chronic Inflammation and Endoscopic Approaches to Diagnosis and Management

**DOI:** 10.3390/medicina62061208

**Published:** 2026-06-22

**Authors:** Sabina Gabriela Luca, Oana Cristina Petrea, Cristina Muzica, Ana Maria Singeap, Ana Maria Buzuleac, Adriana Dunca, Alexandru Sebastian Cotleț, Simona Stefania Juncu, Anca Trifan

**Affiliations:** 1 Department of Gastroenterology, “Grigore T. Popa” University of Medicine and Pharmacy, 700115 Iasi, Romania; sabina-gabriela.luca@d.umfiasi.ro (S.G.L.); anamaria.singeap@yahoo.com (A.M.S.); buzuleac.am@gmail.com (A.M.B.); alexandru-sebastian.cotlet@d.umfiasi.ro (A.S.C.); juncu_simona-stefania@d.umfiasi.ro (S.S.J.); ancatrifan@yahoo.com (A.T.); 2 Institute of Gastroenterology and Hepatology, “St. Spiridon” University Hospital, 700111 Iasi, Romania; 3 Doctoral School, Faculty of Medicine, “Grigore T. Popa” University of Medicine and Pharmacy, Universitatii Street No. 16, 700115 Iasi, Romania; adrianaduncamob@gmail.com

**Keywords:** inflammatory bowel disease, cumulative chronic inflammation, IBD complications

## Abstract

Inflammatory bowel disease (IBD) includes Crohn’s disease (CD) and ulcerative colitis (UC), chronic immune-mediated conditions of the gastrointestinal tract characterized by alternating periods of disease activity and remission with a complex multifactorial pathogenesis. Persistent intestinal inflammation in IBD is a key driver of disease progression and is strongly associated with the development of complications such as dysplasia, colorectal cancer (CRC), intestinal strictures, and fistulas. It may also result in changes in anorectal function, identifiable and classifiable using high-resolution anorectal manometry. Histologic and endoscopic assessments are essential for the evaluation of intestinal inflammation. Cumulative inflammatory burden (CIB) is an important concept that quantifies inflammatory exposure in IBD over time by integrating the severity and duration of histologic inflammation across the disease course, highlighting the importance of long-term inflammatory activity in the development of CRC. Histologic healing may be an important therapeutic target in IBD to reduce the risk of long-term complications. In parallel, emerging precision medicine approaches aim to improve risk stratification and enable early, individualized interventions to reduce disease-related outcomes. Endoscopy also plays a fundamental role in monitoring high-risk patients and guiding treatment decisions. This review aims to characterize the main intestinal complications extending beyond the mucosa that are associated with cumulative chronic inflammation in patients with IBD, including dysplasia, CRC, strictures, fistulas, and anorectal dysfunction in an era increasingly focused on achieving complete mucosal healing. Particular attention is drawn to the significant role of persistent histologic and endoscopic inflammation in disease progression and development of complications, highlighting the specific features and associated risk factors of these disease-related outcomes. Throughout, this review emphasizes the fundamental role of endoscopy in the timely detection, monitoring, and therapeutic management of IBD-related complications, thereby reinforcing its role in comprehensive patient care.

## 1. Introduction

Inflammatory bowel disease (IBD) comprises two entities: Crohn’s disease (CD) and ulcerative colitis (UC) [1,2]. Both are chronic, immune-mediated inflammatory disorders of the gastrointestinal tract with incompletely understood pathogenesis, characterized by a relapsing–remitting disease course [3]. Dysbiosis of the intestinal microbiome is associated with chronic inflammation in IBD and contributes to epithelial barrier dysfunction, intestinal fibrosis, and an increased risk of colorectal carcinogenesis [4].

Persistent and progressively worsening histologic inflammation in patients with IBD is strongly implicated in the development of complications, including dysplasia, which represents an early histopathologic step in the sequence toward colorectal neoplasia. Accurate characterization of disease extent in UC should therefore include both endoscopic and histologic assessments to allow more precise cancer risk stratification. Histological activity predicts the risk of developing neoplastic lesions more reliably than endoscopic inflammation. Beyond disease extension, severity and duration, genetic susceptibility and coexisting conditions, such as primary sclerosing cholangitis (PSC), play a significant role in the pathogenesis of UC-associated colorectal cancer (UC-CRC) [5]. Compared with sporadic cases, UC-CRC exhibits some distinct features in pathogenesis and endoscopic presentation [6]. Likewise, fibrosis leading to stricture formation is a well-recognized complication of CD, particularly within the stenosing phenotype, but its impact has also been recognized in patients with UC [7]. UC has conventionally been considered a disease marked by superficial inflammation with a low propensity for fibrosis. However, a long-standing, fluctuating disease course and a tendency for proximal disease extension can result in fibrosis affecting the full thickness of the intestinal wall. In such cases, the prevalence of strictures may approach that observed in colonic CD [8]. Malignant features can be identified in areas affected by strictures. Recent data indicate that corticosteroid exposure is associated with an increased risk of stricture development, whereas therapy with 5-aminosalicylic acid (5-ASA) appears to confer a lower risk. These associations may reflect underlying treatment indications, with severe and extensive inflammation likely serving as the principal driver of stricture formation, rather than the pharmacologic intervention employed [9].

Current treatment strategies in IBD prioritize mucosal healing [10,11]. Nevertheless, the chronicity of the disease can induce substantial functional gastrointestinal disorders, many of which may remain unrecognized in patients with IBD who are in endoscopic remission, potentially leading to unnecessary diagnostic procedures and inappropriate escalation of therapy [12]. Prolonged inflammation induces structural alterations in the enteric nervous system, including modifications of the interstitial cells of Cajal and enteric glial cells. Notably, such changes have also been documented in histologically normal colonic segments in patients with UC [7]. Nitric oxide (NO), along with nerves releasing vasoactive intestinal peptide (VIP) and substance P, appears to play a critical role in colonic dysmotility [13]. High-resolution anorectal manometry offers a more detailed evaluation of anorectal function, especially anal sphincter pressures and rectoanal coordination in comparison with conventional manometry in patients with IBD [14].

This review highlights the role of cumulative chronic inflammation in the development of intestinal complications extending beyond the mucosa in long-standing IBD, emphasizing associated risk, as well as the importance of endoscopy in diagnosis and therapeutic management. Furthermore, it emphasizes the impact of chronic inflammation on the development of anorectal sensory and motor abnormalities, objectively quantified by high-resolution anorectal manometry, with the aim of improving understanding of the consequences of chronic active disease.

### Methods

A narrative literature review was conducted using PubMed/MEDLINE, Scopus, and Web of Science databases. The search covered seminal and contemporary studies published from 1979 to 2026, with most included studies published in the last decade. Search terms included combinations of “inflammatory bowel disease”, “ulcerative colitis”, “Crohn’s disease”, “chronic inflammation”, “cumulative inflammatory burden”, “inflammatory signaling pathways”, “colorectal cancer”, “dysplasia”, “fistulas”, “strictures”, “anorectal dysfunction”, “anorectal manometry”, “colonoscopy”, “endoscopic surveillance”, “chromoendoscopy”, and “endoscopic management”. Additional relevant articles were identified through manual screening of reference lists. Priority was given to high-quality evidence, including clinical practice guidelines, systematic reviews, meta-analyses, and pivotal original studies.

## 2. Chronic Histological Inflammation as a Central Determinant of UC-CRC

The neutrophilic infiltration of the lamina propria and colonic crypts, resulting in cryptitis and crypt abscesses represents the main histologic indicator of IBD activity [11]. There is evidence indicating that neutrophils, through their functions, can exert both detrimental effects during transepithelial migration in IBD by disrupting the epithelial barrier and beneficial effects through the secretion of cytokines that promote epithelial healing. Additionally, they contribute to the elimination of extracellular microorganisms via phagocytosis, degranulation, and the release of neutrophil extracellular traps, while also participating in the maintenance of mucosal homeostasis through their interactions with other immune cells [15]. Numerous histological indices have been proposed to assess disease activity in UC, some of which are validated for use in clinical practice [16,17]. On the other hand, in CD, a completely validated histological activity score has not yet been established, given the discontinuous nature of the disease, but the Global Histological Activity Score is frequently used [17,18].

The current therapeutic standard aims for deep remission, which, in addition to clinical and biochemical remission, requires complete mucosal healing, including both endoscopic and histological aspects. Given that these two do not always correspond, persistent histological activity, despite endoscopically confirmed mucosal healing, may lead to complications and an unfavorable therapeutic response [11].

The cumulative inflammatory burden (CIB) metric was first described by a research team at St. Mark’s Hospital, led by Chang Ho Ryan Choi [19]. CIB was calculated by taking the average of histological scores between two consecutive colonoscopies and multiplying it by the length of the surveillance interval in years—with the consideration that, for each endoscopic assessment, the maximum histological score observed across all colonic segments was assigned, without averaging segmental scores. The total CIB was subsequently obtained by summing the values across all surveillance intervals. The assumption that disease duration serves as a surrogate for CIB and, consequently, colorectal cancer (CRC) risk is no longer fully supported, particularly in the context of modern therapies capable of maintaining mucosal healing. The severity of disease activity plays a pivotal role in the quantification of CIB: some patients exhibit chronically active and severe inflammation throughout the course of the disease, whereas others experience prolonged periods of remission characterized by minimal inflammatory activity. Furthermore, assessing inflammatory severity solely based on the most recent colonoscopic evaluation provides only a transient snapshot and fails to account for prior inflammatory episodes that may modulate subsequent CRC risk. The study by Choi et al. demonstrated a significant association between CIB and the development of UC-CRC. These findings indicate that patients with persistent histologic inflammation are at an elevated risk of CRC, independent of the episodic severity of inflammation [20].

Therefore, CIB represents an important concept and a promising marker of long-term risk of colorectal neoplasia in UC, by integrating both inflammatory severity and exposure over time, in contrast to assessments based exclusively on disease duration or single time-point evaluations, which reflect only time since diagnosis or inflammatory status at a single moment and fail to capture overall inflammatory exposure and its temporal variability. However, the implementation of this method in current clinical practice remains challenging. As noted by the authors, there are several limitations to the calculation of CIB, as this method is subject to interobserver variability in both endoscopic and histological assessment, is time-consuming, often relies on incomplete endoscopic and histological patient records, and lacks well-standardized histological scoring systems. In addition, inflammatory activity occurring between surveillance examinations cannot be quantified, and the calculation method assumes that the degree of inflammation remains constant throughout each surveillance interval. For a more comprehensive assessment of inflammatory activity over time, CIB could be interpreted in association with other clinical and biological parameters and may contribute to more accurate risk stratification and the individualization of endoscopic surveillance strategies and therapeutic decisions, with the aim of achieving histological healing and reducing the risk of neoplasia in UC [20]. These observations are further supported by the findings of by Gupta et al., which demonstrated that the severity of histologic inflammation in UC is a significant predictor of progression to advanced dysplasia and CRC, with patients experiencing sustained or more pronounced histologic activity over time exhibiting a markedly increased risk of neoplastic transformation [21].

Endoscopic surveillance for CRC is recommended to begin in patients with UC and colonic CD, 8–10 years after disease onset (in the absence of PSC) and CIB may be a more reliable indicator than disease duration for determining the optimal timing of surveillance [5]. A 2025 meta-analysis by Zhang et al. estimated the prevalence of IBD-associated colorectal cancer (IBD-CRC) at 1.54% with a pooled incidence of 1.47 per 1000 person-years [22], closely aligning with the 2014 meta-analysis by Castaño-Milla et al., which reported a CRC incidence of 1.58 per 1000 person-years [23]. In contrast, the landmark meta-analysis by Eaden et al. reported higher values—a CRC incidence of 3 per 1000 person-years and a prevalence of 3.7% among patients with UC [24]. Recent advancements in both medical therapy and endoscopic management of premalignant lesions appear to have lowered the incidence of IBD-CRC [25].

### 2.1. Patient- and Disease-Related Risk Factors in IBD-CRC

In addition to disease severity, there are other risk factors that can lead to the development of IBD-CRC, factors that may be related either to the patient or to the disease itself. Among the patient-related factors are male sex, early age at diagnosis, concomitant PSC, and a family history of CRC, especially in patients with first-degree relatives [26,27]. A study by Söderlund et al. reported that patient gender has a key role in the development of IBD-CRC, with men demonstrating a higher long-term risk relative to women and the mean age at diagnosis being two years higher in women. Men were also more frequently diagnosed with proximal colon and rectal cancers [28]. Furthermore, the cumulative evidence from 60 studies revealed that the risk of IBD-CRC was lower in female patients compared to male patients [29]. A plausible explanation could be the protective effect of estrogen on inflammatory processes [26,27,28]. The diagnosis of UC at a young age has also been associated with an increased risk of developing CRC [30], which may be explained by an inadequate control of intestinal inflammation with relatively persistent and severe disease activity, creating the potential for disease complications, particularly in patients diagnosed with IBD before the age of 30 [31]. Ekbom et al. demonstrated that an age younger than 15 years at diagnosis of IBD was an independent risk factor for progression to CRC [32,33], contrary to Wu et al.’s study, which showed that the risk of CRC increases with older age at the diagnosis of IBD, probably due to the accumulation of mutations throughout life, before the onset of IBD [32], data comparable to those reported in the studies by Greenstein et al., who reported that the cumulative probability of developing CRC reached 64% at 40 years, significantly higher than 34% at 30 years [34]. The age at diagnosis of CRC in patients with IBD is lower than in the general population without IBD, as demonstrated by a study conducted by Neri et al., which found that almost a quarter of patients under the age of 40 were diagnosed with CRC, while more than one third of cases occurred in patients younger than 50 years [35]. Patients with IBD, especially those with UC and concomitant PSC, have a higher risk of developing CRC [36]. A meta-analysis conducted by Zheng et al. showed that patients with both conditions were at an increased risk of dysplasia and CRC compared with patients without concomitant PSC (OR 3.24; 95% CI 2.14–4.90) [37]. Therefore, there is a strong association between PSC and IBD. It remains to be determined whether PSC represents only an extraintestinal manifestation or a distinct disease entity with similar pathogenic pathways [36,38]. Epidemiological data suggest that PSC and IBD may correspond to a single condition, as they are diagnosed simultaneously in most cases and exhibit a similar geographical distribution, with a higher incidence reported in North America and Northern Europe [38,39,40]. Colonic inflammation in UC patients with PSC presents some distinct features compared with UC alone, being more extensive and showing much more severe histological activity in the right colon, with a gradual decrease toward the descending colon. This inflammatory phenotype observed in the right colon may also be driven by elevated bile acid concentrations in the proximal colon, which cannot be properly absorbed due to cholestasis and therefore cause additional inflammatory lesions, leading to dysplastic changes and CRC. This condition may be associated with backwash ileitis caused by inflammatory dysfunction of the ileocecal valve and inflammatory rectal sparing. Currently, there is no uniformly accepted definition for rectal sparing, but it is characterized by complete mucosal healing in the absence of topical therapy over the previous 6 months [38,41]. In patients with CD associated with PSC, colonic involvement predominates in the gastrointestinal tract, transmural manifestations are less frequent, and the disease course is milder than in UC [40]. First-degree relatives of individuals diagnosed with sporadic CRC have an increased risk of developing CRC themselves [42,43,44]. Hence, family history of CRC is also considered an important patient-dependent risk factor for developing IBD-CRC [26,27]. A study led by Askling et al. reported that patients with IBD who had a family history of CRC had a twofold higher overall risk of CRC (RR ≈ 2.5; 95% CI 1.4–4.4). The risk was markedly higher (RR 9.2; 95% CI 3.7–23) if a first-degree relative was diagnosed before the age of 50 [45]. In addition, a meta-analysis based on 15 studies, also reported an increased risk of CRC in patients with IBD and a positive family history of CRC (OR 2.62; 95% CI 1.93–3.57) [29]. Therefore, family history could be a valuable tool for initiating intensive colonoscopy screening in this category of patients [45].

Disease-related factors include previously described disease duration and histological severity, as well as disease extent, degree of dysplasia, and the presence of stenoses and pseudopolyps [26,27]. Regarding disease extent, the highest risk of CRC is associated with left-sided and extensive colitis, whereas proctitis is associated with a minimal risk [46]. Colonic involvement in CD increases the risk of developing CRC, chronic inflammation playing a central role, though the mechanisms of pathogenesis are incompletely understood. CD-associated CRC necessitates a differential diagnosis with sporadic CRC and Lynch syndrome [47].

### 2.2. Role of Dysplasia and Genetic Alterations in IBD-CRC Development

Chronic inflammation associated with IBD produces oxidative stress, DNA alterations and contributes to the development of dysplasia, which is considered one of the most important risk factors for CRC progression. There are significant differences between dysplastic lesions in UC and those in sporadic cancers, both from genetic and macroscopic perspectives [5]. The progression from no dysplasia through indefinite dysplasia, low-grade dysplasia (LGD), and high-grade dysplasia (HGD) represents the stages of carcinogenesis encountered in IBD before the development of CRC, although these stages may be absent in clinical practice. IBD-CRC can arise through genomic instability, including both chromosomal and microsatellite instability (MSI), as well as through the CpG island methylation phenotype [48,49]. Aneuploidy is considered a marker of chromosomal genomic instability [5,48] and is characterized by abnormal DNA content resulting from aberrant chromosome segregation, and is frequently associated with loss of heterozygosity and inactivation of tumor suppressor genes [48]. In a study conducted by Rubin et al., flow cytometric analysis of colonic tissue demonstrated that aneuploidy could precede dysplasia, and, in some cases, persist or expand over time in UC patients [50]. These findings may support the concept of field carcinogenesis in UC and partly explain the higher incidence of synchronous and metachronous UC-CRC compared with sporadic CRC [5]. The theory that gradual development from dysplasia to carcinoma may be correlated with a reduction in excess genetic material was also supported in the study conducted by Rubin et al., as the average DNA content in aneuploid biopsy samples from specimens with colonic cancer was markedly reduced compared with that in dysplastic samples [50]. Likewise, in a study by Tsai et al., flow cytometry was used to evaluate the impact of colonic aneuploidy in IBD patients with flat LGD, revealing a higher risk of progression to HGD or CRC in those with LGD and aneuploidy compared with the cohort exhibiting LGD and normal DNA content. At 1 year, the detection rate of HGD or CRC among subjects with flat LGD and aneuploidy was 57.3% (95% CI 23.9–81.7%), increasing to 100% at 12 years (95% CI 24.4–100%), whereas individuals with flat LGD and normal DNA content had a 1-year detection rate of 4.6% (*p* < 0.001) and a 12-year detection rate of 33.2% (*p* = 0.001) [51]. Flow cytometry may be useful for risk stratification and may complement clinical decision-making regarding early colectomy or increased colonoscopic surveillance [50,51].

Dysplastic lesions in IBD can be histologically subdivided into two types: conventional, which is the most well-documented in the literature, and unconventional, which comprises a minimum of six atypical histological variants. The most recognized variant of unconventional dysplasia is the hyper-mucinous form, characterized by a tubule-villous or villous architecture with prominent mucinous differentiation representing more than 50% of the lesion, frequently exhibiting aneuploidy. Unconventional dysplasia is common in patients with IBD-CRC, it is more often associated with poorly differentiated CRC and can coexist with conventional dysplasia [52]. Moreover, it is typically located adjacent to CRC or in the same colonic segment and carries a comparable risk of progression to CRC as conventional lesions [52,53]. Other histological subtypes of unconventional dysplasia that may be encountered include goblet cell deficient, terminal epithelial differentiation, traditional serrated adenoma, sessile serrated lesion, and serrated lesion, not otherwise specified, all of which represent diagnostic challenges for pathologists [52].

There are similarities in the genetic mutations that occur in UC-CRC and sporadic CRC, but the temporal sequence of mutations differs. Hence, genetic alterations in the adenomatous polyposis coli (APC) and Kirsten rat sarcoma virus (KRAS) genes are detected less frequently in UC-CRC patients and tend to arise later in the disease course. By contrast, in sporadic CRC, APC mutations are observed early in carcinogenesis. Genetic mutations in the tumor protein p53 (TP53) gene—accompanied by downregulation of TP53 expression—and amplifications of the MYC proto-oncogene represent early events in carcinogenesis in patients with IBD. On the other hand, in sporadic cancer, loss of TP53 gene function is considered a late event and plays a crucial role in adenoma to carcinoma transition. An increase in TP53 mutation has been observed in the mucosa of patients with active UC, in the absence of CRC, suggesting that chronic inflammation predisposes to the occurrence of these genetic modifications at an early stage [27,48,49,54].

While the majority of UC-CRC develop through the chromosomal instability pathway, a smaller proportion, approximately 15%, arise through the MSI pathway, like Lynch syndrome. MSI results from loss of function of genes involved in DNA base-pair mismatch repair during DNA replication, including human MutL homolog 1 (MLH1) and human MutS homolog 2 (MSH2). This loss promotes genomic instability and contributes to the development of CRC. MSI can be subdivided into high (MSI-H) or low (MSI-L) microsatellite instability, depending on the number of unstable genetic markers identified [48].

Epigenetic alterations contribute to colon carcinogenesis in UC through promoter hypermethylation, leading to gene silencing [49]. Hypermethylation of the p16INK4a promoter region is a frequent and early event in the dysplasia to CRC sequence associated with UC. The frequency of p16 promoter hypermethylation in colonic biopsies increases with the degree of dysplasia and is present in 100% of cancer samples [48,55]. Additionally, an early phase in the carcinogenesis of a subgroup of patients with IBD-CRC is hypermethylation of the MLH1 promoter, which can also be detected in nondysplastic mucosa and is strongly associated with MSI-H tumors. Hence, hypermethylation and microsatellite instability may be part of the same tumor process [49,56,57].

### 2.3. The Main Proinflammatory Pathways in the Initiation and Progression of IBD-CRC

Inflammatory signaling pathways, such as cyclooxygenase-2 (COX-2)/prostaglandin E2 (PGE2), nuclear factor kappa B (NF-κB), interleukin-6 (IL-6)/signal transducer and activator of transcription 3 (STAT3), Interleukin-23 (IL-23)/T helper 17 cells (Th17), and transforming growth factor beta (TGF-β)/SMAD, play a key role in the development of IBD-CRC by regulating the expression of various inflammatory mediators and contributing to the formation of a favorable environment for carcinogenesis [58].

#### 2.3.1. COX-2/PGE2-Mediated Mechanism

Increased levels of COX-2 were observed in both IBD-CRC and sporadic CRC [58,59,60]. COX-2 is an inducible enzyme that is usually absent in healthy tissues but becomes expressed following the onset of inflammatory conditions [58]. Notably, increased COX-2 expression has been observed in approximately 85% of sporadic CRC cases [58,59,61,62]. In inflamed colonic segments affected by IBD, pro-inflammatory cytokines such as interleukin-1 (IL-1), interferon-gamma (IFN-γ) and tumor necrosis factor-alpha (TNF-α) induce COX-2 expression [49], leading to increased prostaglandin production, such as PGE2 [58,59,60]. Accumulating evidence indicates that PGE2 appears to contribute to the processes of carcinogenesis, such as cell proliferation, angiogenesis, and resistance to apoptosis in CRC and IBD-CRC [58,59,60,63]. PGE2 binds to prostaglandin E receptors, including EP2 and EP4, to exert pathogenic activity. Signaling through EP2 and EP4 increases cyclic adenosine monophosphate levels, which has an immunosuppressive effect and facilitates tumor pathophysiology [58,59]. EP4 is thought to have a fundamental function in epithelial survival and regeneration by activating anti-apoptotic and proliferative signaling pathways, and it is believed to mediate the pro-oncogenic effects of PGE2 in CRC. PGE2 may also facilitate the association between colonic carcinogenesis and chronic inflammation through a self-amplifying loop involving peroxisome proliferator-activated receptor gamma (PPARδ) and the COX-2/PGE2 pathway [58]. The risk of developing CRC may decrease by 40–50% following long-term use of anti-inflammatory drugs (NSAIDs) [49,58,64]. There is more data on the role of NSAIDs, especially aspirin, in the prophylaxis of both sporadic CRC and IBD-CRC, but they are not used yet in routine clinical practice for this purpose [65,66,67]. Among selective COX-2 inhibitors, some of which can cause significant cardiovascular side effects [68,69], celecoxib may provide a safer option for CRC prophylaxis in selected individuals. More selective pharmacological inhibition of PGE2 production downstream of COX-2 could improve efficacy while decreasing adverse reactions. Further evaluation of PGE2 receptor antagonists and PGE2 synthase inhibitors is essential, with the aim of providing CRC prophylaxis without causing significant adverse effects [56,60]. For example, PPARδ is one of the downstream targets of the COX-2/PGE2 pathway that could be a future therapeutic target [58]. COX-2 could be used in the future as a biomarker for the diagnosis of CRC, even at early stages [59].

#### 2.3.2. Nuclear Factor Kappa B (NF-κB)-Mediated Mechanism

There is evidence supporting the involvement of the NF-κB pathway in the pathogenesis of UC-CRC in animal studies, as exemplified by the research of Greten et al. [70].

A group of transcription factors, referred to as NF-κB, includes five members: RelA (p65), RelB, c-Rel, NF-κB1 (p50), and NF-κB2 (p52). Each member of the NF-κB family exhibits common structural features and contains a conserved domain, the Rel homology domain, which is involved in dimerization, nuclear localization, DNA binding and interaction with the inhibitory protein IκB. NF-κB acts through two distinct pathways: the canonical and non-canonical pathways [71,72,73]. In both pathways, NF-κB is retained in the cytoplasm in a complex with its inhibitor [74].

Concerning the canonical pathway, the inhibitory protein IκB retains NF-κB in the cytoplasm as an inactive complex. Multiple inflammatory factors, such as the cytokines TNF-α and IL-1β, and the bacterial component lipopolysaccharide induce dissociation of the NF-κB–IκB complex [58,71]. Therefore, activation of the canonical pathway leads to the activation of the IκB kinase (IKK) complex (IKKα, IKKβ and NEMO IKKγ), which, once activated, causes the degradation of the IκB proteins, thus allowing the released NF-κB (usually p50/RelA) to translocate into the nucleus and transactivate target genes, such as proinflammatory genes [71,74]. The canonical NF-κB pathway, activated upon exposure to external inflammatory stimuli, modulates immune and inflammatory responses, cell proliferation and differentiation, as well as cell survival [72].

On the other hand, the non-canonical NF-κB pathway is activated by several TNF family receptors, including B-cell activating factor receptor (BAFF-R), CD40 and lymphotoxin β receptor (LTβR) and contributes to the development of certain immune cell lineages [75]. It also seems to play a significant role in the formation of tertiary lymphoid organs and participates in chronic inflammation and related diseases [71]. NF-κB-inducing kinase (NIK) has a key role in the non-canonical pathway. TNF receptor-associated factor 3 (TRAF3) binds to the N-terminal region of NIK, targeting it for proteasomal degradation [75]. The E3 ubiquitin ligase cIAP degrades TRAF3, leading to the accumulation of NIK upon stimulation (e.g., via BAFF-R). NIK then activates IKKα, which phosphorylates p100. Processing of p100 to p52, which functions as an IκB-like protein, releases the RelB/p52 dimer for nuclear translocation and transcriptional activation of target genes [72]. Genes encoding antiapoptotic regulators, including GADD45β (growth arrest and DNA-damage-inducible 45β), BFL1 (BCL-2–related protein), and BCL-XL (B-cell lymphoma XL), are recognized as target genes of the NF-κB pathway. In patients with IBD, upregulation and NF-κB activation were identified in macrophages and epithelial lining cells in the mucosa, which was associated with elevated levels of proinflammatory cytokines including TNF-α, IL-1, and IL-6 [58].

#### 2.3.3. IL-6/STAT3-Mediated Mechanism

The important role of IL-6 in patients with IBD is increasingly recognized, with its levels correlating with the severity of inflammation and being associated with the development of colorectal neoplasia. Binding of IL-6 to its membrane-bound receptor, interleukin-6 receptor (IL-6R), and formation of the IL-6/IL-6R complex initiates the classical IL-6 signaling pathway, activating intracellular pathways such as Janus kinase/signal transducer and activator of transcription (JAK/STAT). IL-6 can also bind to a soluble form of its receptor, soluble interleukin-6 receptor (sIL-6R), forming an IL-6/sIL-6R complex, which then interacts with glycoprotein 130 (gp130) in cells that do not express IL-6R but do express gp130, thereby initiating intracellular signaling. In IBD, STAT3 activation, mediated by the interaction between IL-6/sIL-6R complexes and gp130, leads to increased expression of anti-apoptotic factors [58,76,77].

#### 2.3.4. IL-23/Th17-Mediated Mechanism

Interleukin-17 (IL-17)-producing Th17, key components of the IL-23/Th17 axis, are implicated in the pathogenesis of IBD, contributing both to the maintenance of intestinal inflammation and to the development of IBD-CRC. IL-17A, the main effector cytokine produced by Th17 cells, promotes intestinal inflammation and angiogenesis by stimulating the production of vascular endothelial growth factor. In addition, Th17 cells secrete additional cytokines, such as Interleukin- 21 (IL-21) and Interleukin-22 (IL-22), which contribute to tumor progression by amplifying the inflammatory response and promoting cellular proliferation. IL-23 is an essential regulator of the Th17 response, being involved in the stabilization and expansion of these cells. Furthermore, TGF-β and IL-6 promote the expression of the IL-23 receptor (IL23R) on Th17 cells, thereby enhancing the biological effects of IL-23. Experimental studies have shown that blockade of the IL-23 signaling pathway through the use of an anti-IL-23p19 monoclonal antibody induces Th17 cell apoptosis and reduces the severity of colitis, highlighting the important role of the IL-23/Th17 axis in the pathogenesis of IBD [58,78].

#### 2.3.5. TGF- β-Mediated Mechanism

TGF-β is involved in maintaining intestinal homeostasis by modulating the functions of immune cells, epithelial cells, and the luminal microbiota, which are key factors in the pathogenesis of the disease. Activated TGF-β binds to the type II TGF-β receptor (TGFβRII), after which a complex is formed with the type I TGF-β receptor (TGFβRI). The resulting receptor complex activates intracellular signaling through Smad-dependent canonical pathways (Smad2 and Smad3 phosphorylated form a complex with Smad4 and translocate to the nucleus to regulate target gene transcription) and Smad-independent non-canonical pathways. Smad7 is an intracellular inhibitor of TGF-β signaling (I-Smad), which binds to TGFβRI and acts as a negative feedback mechanism that inhibits canonical TGF-β signaling. Patients with IBD exhibit elevated levels of Smad7, which leads to reduced Smad3 phosphorylation and results in impaired TGF-β signaling, a mechanism considered to be potentially involved in the pathogenesis of IBD [79]. Hence, chronic inflammatory signaling pathways, including COX-2/PGE2, NF-κB, IL-6/STAT3, IL-23/Th17, TGF-β appear to play a central role in linking inflammation to intestinal epithelial barrier dysfunction and impaired mucosal repair, thereby contributing to a pro-tumorigenic microenvironment that promotes IBD-CRC [58,74].

#### 2.3.6. Multiple microRNAs (miRNAs)

miRNAs, a type of short non-coding RNA encoded within the human genome and transcribed by RNA polymerase II, are subsequently processed through Drosha- and Dicer-dependent pathways, including nuclear processing and Exportin-5/RanGTP-mediated cytoplasmic export, to generate mature regulatory RNAs. These molecules are involved in the pathogenesis of IBD, playing a role in complex regulatory networks of the gut microbiome, intestinal integrity, and intestinal inflammatory processes, and are implicated in the progression of immune-mediated inflammatory diseases through the regulation of cell cycle and homeostasis, cellular survival, differentiation, proliferation, and autophagy. A potential future therapeutic strategy may involve inhibition of miRNA activity, but further studies are required [80]. The pathogenesis of IBD is driven by both classical inflammatory pathways, including COX-2/PGE2, NF-κB, IL-23/Th17, and IL-6/STAT3 signaling, as well as post-transcriptional regulatory mechanisms mediated by microRNAs, which together form interconnected regulatory networks that coordinate immune responses, epithelial homeostasis, and intestinal barrier integrity Among these, miRNAs such as miR-21 and miR-146a play an important role in modulating inflammatory signaling pathways, immune cell activation, epithelial homeostasis, and neoplastic progression within the intestinal microenvironment [58,80].

Thus, chronic inflammation in IBD is associated with disruption of the intestinal epithelial barrier through epithelial damage, leading to increased intestinal permeability and translocation of microbial antigens, thereby further amplifying the inflammatory response. In this context, aberrant repair processes and cellular proliferation may promote the persistence of inflammation and the formation of a pro-tumorigenic microenvironment, which indirectly contributes to the progression toward IBD-CRC. Recent data highlight the importance of restoring intestinal epithelial barrier integrity through modulation of inflammatory signaling as a therapeutic strategy in UC. As an example, Lv et al. identified vanillin as a bioactive compound capable of directly targeting the serine 302 (Ser302) residue of β-arrestin 1 (Arrb1), thereby inhibiting NF-κB activation and contributing to the maintenance of epithelial barrier integrity [74,79,81].

#### 2.3.7. Emerging Future Directions

Another challenge in the implementation of precision medicine in current clinical practice is the identification and validation of biomarkers in IBD, as well as their potential to guide disease management and predict treatment response, an area that continues to evolve rapidly. In addition to established biomarkers that have become indispensable in IBD management, such as fecal calprotectin and C-reactive protein (CRP), numerous emerging biomarkers are currently under investigation, including miRNAs, Oncostatin M (OSM)—a cytokine belonging to the IL-6 family, and B-cell Activating Factor (BAFF)—a cytokine of the TNF superfamily, as well as other biomarkers derived from the fields of proteomics, genetics, and metabolomics [82]. In a study conducted by Medhat et al., serum levels of TNF-α and IL-12/23 (p40) were measured in patients with IBD before and after the initiation of biologic therapy, and these inflammatory profiles were correlated with clinical response to treatment. The results showed that patients with a predominance of TNF-α–mediated signaling exhibited a superior response to anti-TNF-α therapy, suggesting that cytokine profiling may represent a useful tool for personalizing therapeutic strategies in IBD [83].

### 2.4. Therapeutic and Pharmacological Agents and Their Promising Protective Effects on Dysplasia and CRC in IBD Patients

#### 2.4.1. 5-ASA Therapy

5-ASA, used in the treatment of mild to moderate UC, shares structural similarity with aspirin and has been suggested to exert chemopreventive effects in the development of CRC by modulation of local colonic inflammation [84]. 5-ASA appears to protect cells against free radical damage by decreasing NO synthase activity, thereby reducing reactive oxygen species, while also modulating the Wnt/β-catenin pathway, upregulating PPAR-γ, and inhibiting NF-κB, thereby suppressing tumor growth through both COX-2–dependent and COX-2–independent mechanisms [85]. Mesalazine is one of the most common therapies used in UC [86]. The efficacy of mesalazine in reducing the risk of CRC in patients with UC has shown variable and sometimes contradictory results across different studies [87]. The analysis by Bonovas et al. indicated an association between 5-ASA use and an overall reduced risk of CRC in patients with IBD (RR 0.57; 95% CI 0.45–0.71). 5-ASA therapy was also associated with a reduced risk of both cancer (RR 0.58; 95% CI 0.45–0.74) and dysplasia (RR 0.54; 95% CI 0.35–0.84), mainly due to mesalazine rather than sulfasalazine. Moreover, CRC risk reduction appeared more pronounced in UC than in CD (RR 0.50; 95% CI 0.38–0.64 vs. RR 0.76; 95% CI 0.43–1.33) [88].

A sub-analysis within a meta-analysis by Qiu et al. reported odds ratios for CRC risk in UC patients receiving 5-ASA therapy (OR 0.40; 95% CI 0.30–0.55), but found no significant reduction in the risk of dysplasia (OR 0.18; 95% CI 0.02–1.53) or the composite outcome of CRC and dysplasia (OR 0.62; 95% CI 0.36–1.06). In patients with CD, 5-ASA was not associated with a reduced risk of CRC or dysplasia in population-based and clinical studies (population-based OR 0.37; 95% CI 0.12–1.14; clinical OR 0.73; 95% CI 0.45–1.19). Nevertheless, separate assessments of CRC and dysplasia risks in this patient’s category showed a potential preventive effect of 5-ASA on CRC only, although the data are limited [89]. Also, doses ≥1.2 g/day of mesalazine were associated with a stronger protective effects compared to lower doses in the study by Qiu et al. [89]. These findings agree with those of Zhao et al. and Eaden et al., who reported that mesalamine ≥1.2 g/day exerts a chemopreventive effect in UC patients [90,91]. However, in the study by Zhao et al., the use of 5-ASA for the prevention of CRC in patients with extensive UC conferred little benefit [90].

In addition, 5-ASA seemed to confer a greater protective effect in Asian patients with IBD, followed by European patients, whereas the less pronounced effect observed in North American patients may be explained by interactions with environmental and genetic factors in this population [89,90].

#### 2.4.2. Immunomodulators

Azathioprine (AZA) and mercaptopurine (MP) are antimetabolites administered as immunosuppressive prodrugs that belong to the thiopurine class [92]. Thiopurines, used to maintain remission in patients with CD and in patients with UC who are unable to tolerate 5-ASA or require repeated courses of corticosteroids [93,94], have been associated with a protective effect against the development of IBD associated CRC, despite their known risk of extraintestinal malignancies [86]. In a meta-analysis conducted by Lu et al., which analyzed 24 studies, an association between thiopurine use and a reduced risk of CRC was observed in patients with UC (OR 0.67; 95% CI 0.45–0.98), but not in patients with CD (OR 1.06; 95% CI 0.54–2.09), as about 30% of CD patients have no colonic involvement, which according to the authors, may underestimate the overall protective effect of thiopurines in CD. Thiopurines were linked to a significant reduction in the risk of CRC (OR 0.65; 95% CI 0.45–0.96) and advanced colorectal neoplasia (CRC/HGD) (OR 0.62, 95% CI 0.44–0.89), but not in the risk of dysplasia (OR 0.90; 95% CI 0.37–2.21), although the preventive action of thiopurines in reducing the risk of dysplasia could be underestimated, given that UC patients may have had previously undiagnosed colonic dysplasia before starting thiopurine treatment. The doses considered to have the optimal efficacy are 2.0–2.5 mg/kg for AZA and 1.0–1.5 mg/kg for MP. Like 5-ASA, geographic variability in the neoplastic protection conferred by thiopurines was observed, with a protective role identified in studies from Europe, but not in those from North America, Asia, or Africa [92]. Another study by Zeng et al. did not demonstrate a protective effect of thiopurines on the risk of progression from LGD to advanced colorectal neoplasia in patients with IBD. The authors noted that the concomitant presence of PSC could influence the potential chemopreventive effects of thiopurines and 5-ASA, but the results did not reach statistical significance, and further studies are warranted [95].

#### 2.4.3. ANTI-TNF-α Agents

The ECCO guideline recommends the use of anti-TNF-α agents in patients with UC and CD with moderate-to-severe disease activity who do not respond to or cannot tolerate conventional therapy, for both induction and maintenance of remission [96,97,98]. The findings of a French national population-based cohort study revealed that anti-TNF-α therapy is associated with a reduced risk of CRC in patients with long-standing UC (≥10 years) [99]. Furthermore, results from a preclinical study in a mouse model of colitis-associated CRC suggested that modulation of TNF-α signaling may constitute a therapeutic strategy for CRC in patients with UC [100].

#### 2.4.4. Statins

Statins, in addition to their cholesterol-lowering role mediated through inhibition of 3-hydroxy-3-methyl-glutaryl-coenzyme A reductase (HMGCR), also reduce the production of other compounds indispensable for the modification and activation of cellular proteins involved in the development of tumors, such as members of the Ras/Rho superfamily [86,101,102]. Statins may contribute to the reduction in polyps and distant tumor dissemination. Their antioxidant effects and ability to inhibit cell adhesion, angiogenesis, and inflammation are achieved independently of HMGCR [86,101,103]. One of the first studies to evaluate the association between statin use and the risk of CRC in patients with IBD was conducted by Samadder et al. This study revealed that prolonged statin therapy was associated with a reduced risk of both sporadic CRC (OR 0.49; 95% CI 0.39–0.62) and IBD-associated CRC (OR 0.07; 95% CI 0.01–0.78) [104]. Additionally, in an observational cohort study, Ananthakrishnan et al. indicated that statin use was associated with a significantly decreased risk of CRC in patients with IBD (OR 0.42; 95% CI 0.28–0.62), even though the authors emphasized the limitations of the study and the necessity for further research to clarify the potential chemopreventive effect of statins in this patient population [103]. On the other hand, other data have not demonstrated a potential benefit of statins in the chemoprevention of CRC in patients with IBD [105].

Therefore, several pharmacological agents used in the management of IBD, including 5-ASA, immunomodulators, and anti-TNF-α agents, as well as non-IBD therapies such as statins, have been investigated for a potential association with a reduced risk of CRC. The most consistent evidence has been reported for 5-ASA, whereas data for immunomodulators, anti-TNF-α agents, and statins are primarily derived from observational studies with heterogeneous results. Furthermore studies are required to clarify their potential chemopreventive role [84,88,90,92,99,103,105].

## 3. Endoscopy in the Detection of Neoplastic Lesions in IBD

Endoscopy is an essential tool for the detection, monitoring, and therapeutic management of neoplastic lesions associated with IBD [106,107]. Endoscopic surveillance programs, together with mucosal healing, are associated with a reduced incidence of IBD-CRC. Although mucosal healing is currently a major therapeutic target, adherence to surveillance recommendations remains suboptimal, despite increasing knowledge regarding the long-term benefits of colonoscopic monitoring, which include early detection of neoplastic lesions and improved prognosis. In a study conducted by Ballester et al., the use of surveillance colonoscopy was associated with a 5–6-fold increase in the likelihood of detecting dysplastic lesions or CRC in early stages. In addition, the authors showed that 86% of patients with an indication for CRC screening underwent surveillance colonoscopy. Recommended intervals were not always respected, and full adherence to ECCO guidelines was observed in only 27% of cases [108]. Suboptimal adherence to guideline recommendations has also been reported in an Australian study, where the authors found that 70% of patients underwent their first surveillance colonoscopy at the correct interval, while only 37% maintained adherence to the recommended timing of follow-up colonoscopies according to guidelines, and the use of chromoendoscopy was also limited, being performed in only 16% of patients [109].

The use of high-definition white-light endoscopy (HD-WLE), with or without dye-based chromoendoscopy (DCE), has improved the detection of neoplastic lesions compared with standard-definition white-light endoscopy (SD-WLE). In DCE, indigo carmine or methylene blue are used, applied during withdrawal of the colonoscope over the entire surface of the colonic mucosa. Thus, the margins of neoplastic lesions are highlighted, allowing more precise delineation. Currently, the use of HD-WLE is recommended for dysplasia detection. In addition, DCE is preferred over SD-WLE, demonstrating a higher lesion detection rate. When HD-WLE is used in combination with DCE, targeted biopsies are recommended from irregular lesions and from areas with previously invisible dysplasia. Virtual chromoendoscopy (VCE), with similar efficacy to DCE in the diagnosis of IBD-associated neoplasia, utilizes narrow-band imaging (NBI) and highlights the surface architecture and vascularization of mucosal lesions [110]. SD-WLE was the main surveillance method in patients with IBD and was based on random four-quadrant biopsies every 10 cm along the colon for dysplasia detection. Currently, DCE with targeted biopsies has become the method of choice due to its superior efficacy and cost-effectiveness compared with SD-WLE, although the introduction of HD-WLE has significantly improved surveillance accuracy [108,111]. In several studies, HD-WLE has demonstrated a detection capability comparable to DCE, which has led to an increasing use of this technique for identifying neoplastic lesions [112]. In a meta-analysis, HD-WLE and DCE combined (HD-WLE-DCE) represented the endoscopic modality with the best overall performance, with a relatively modest benefit in dysplasia detection compared with HD-WLE [113]. Confocal laser endomicroscopy (CLE) and endocytoscopy (EC), using fluorescent agents and methylene blue respectively, allow real-time endoscopic evaluation of colonic histology, with EC providing a more detailed assessment of crypt architecture and nuclear morphology. Their availability in routine clinical practice remains limited. Molecular endoscopy (ME) represents a potential future technique for real-time dysplasia surveillance. It uses advanced imaging combined with targeted molecular probes to detect specific biomarkers in the gastrointestinal mucosa. Fluorescently labelled antibodies or peptides are used to bind dysplasia-associated proteins [111].

In clinical practice, the widespread use of DCE remains limited due to longer procedure time, the need for additional training, and dependence on endoscopist expertise. Nevertheless, major international guidelines continue to recommend DCE and targeted biopsies for dysplasia surveillance in patients with IBD and discourage routine use of random biopsies, reserving this approach for selected high-risk groups such as patients with PSC. Endoscopic surveillance should be initiated 8 years after disease onset in patients with extensive UC or CD with significant colonic involvement, according to risk stratification, with intervals adapted to risk: 1–3 years in high-risk patients (active inflammation, history of dysplasia, or family history of CRC) and up to 5 years in low-risk patients [111]. A summary of endoscopic surveillance techniques for IBD-associated neoplasia and their clinical applicability in its detection is presented in Table 1 [108,109,111,112,113].

Artificial intelligence (AI) represents a novel tool that contributes to the standardization of endoscopic assessment, both in the evaluation of disease activity and in the real-time calculation of endoscopic scores. Furthermore, it has demonstrated its utility in the detection of colorectal neoplasia through the development of specific applications for the surveillance of patients with IBD. Moreover, to reduce interobserver variability in the assessment of histological disease activity through the automated analysis of biopsies from UC patients, AI systems could contribute significantly to the standardization of evaluation, with promising results already being reported. It represents a promising tool not only for predicting the course of IBD and for evaluating the response to biological therapies, but also for predicting the risk of clinical relapse in patients with UC in clinical remission, as well as for the development of novel therapeutic strategies, supporting the implementation of precision medicine [114,115].

## 4. Stenosis in IBD

### 4.1. Stenosis in CD

CD can modify its clinical behavior over time, evolving from an inflammatory phenotype to a stenosing or penetrating one with possible perianal involvement [116]. Hence, this characterization of disease behavior in CD—such as inflammatory, stenosing, and penetrating—is rather limiting, given the increasing evidence for the progressive nature of the disease and the concept of chronic accumulation of inflammatory intestinal lesions, leading to complications such as strictures, fistulas, and abscesses [117]. Stenoses in CD are more frequently the result of a combination of inflammatory processes and fibrosis, which develops following excessive extracellular matrix accumulation driven by long-term inflammation [116]. Magnetic resonance imaging (MRI) may be part of a comprehensive assessment used to support the differentiation between inflammatory and fibrotic intestinal strictures [118].

In a study, Beaugerie et al. observed that, in patients with CD, age under 40 at diagnosis, use of corticosteroids at the first flare, and the presence of perianal disease at disease onset were independently correlated with an increased risk of a debilitating clinical course and, consequently, with impaired quality of life during the five years following diagnosis [119]. Moreover, age at diagnosis was correlated with disease extent in a study by Lapidus et al. Late onset of disease was associated with more frequent left-sided colon involvement and also with a lower propensity for surgery, whereas complete colon involvement was associated with younger ages at diagnosis and increased need for surgery [120]. In patients with CD and a long course of the disease (10 years from diagnosis), the cumulative incidence of complications was estimated at 69–70%, and the presence of a stricture was observed in approximately 50% of patients [117]. The small intestine is the site where CD stenoses are most common, whereas stenoses in the left colon are less frequent and are linked to precancerous lesions [116,121]. A relative risk of 3.2 (95% CI 2.7–3.6) for surgery was reported in patients with ileocecal CD, compared with patients with isolated colorectal disease. A similar relative risk was also observed in patients with isolated small bowel disease. Consequently, an apparent association exists between the location and extent of disease and the need for surgery [122]. In a recent meta-analysis, prior ileocolic resection, disease at the ileocolic resection site, perianal disease, smoking, and the presence of scattered ulcers at the anastomosis were recognized as independent predictors of early post-surgical recurrence. Ileocecal resection may predispose to disease recurrence, given that ileal involvement is unlikely to remain confined and may extend to other segments of the small intestine [123].

### 4.2. Stenosis in UC

A clinically significant intestinal stricture is characterized by narrowing of the lumen, which causes obstruction, along with concomitant dilation of the proximal intestinal segment [9]. Even though colonic strictures are less common in UC patients compared to those with CD, they have been observed in an estimated 5% of UC patients [124]. In a study analyzing data from 439 patients with UC over a 10-year period, a 3.6% incidence of colorectal strictures was reported, a decrease compared to previous studies, interpreted in the context of current treatments and therapeutic strategies. The authors reported rates of 42.9% for dysplasia and 33.3% for CRC in patients with strictures, higher percentages compared to previous studies [9]. Xu et al. reported that colorectal stricture in patients with UC was associated with lower remission rates, a higher number of complications, including the development of CRC, and, furthermore, an increased need for surgical treatment. Colorectal stricture was identified as the most significant risk factor for malignant transformation (OR 9.35; 95% CI 2.84–30.76; *p* < 0.001). Additionally, the authors demonstrated a significant association between disease duration, moderate anemia, the presence of PSC and the development of colorectal stricture [125]. The evidence is also supported by the study of Choi et al., which found that colonic stricture and the tubular appearance of the colon, reduced in size as an expression of long-term persistent disease, represent independent risk factors for the occurrence of CRC [20].

### 4.3. Endoscopic Management of Colonic Strictures in Patients with IBD

Biopsies of strictures in IBD are mandatory before endoscopic treatment. Fibrotic or mixed (fibrotic and ulcerated) strictures could be considered indications for endoscopic therapy. The length, location of the stricture, and the extent of pre-stricture dilation in CD guide the choice of therapy, whether endoscopic or surgical. Hence, strictures with a length >4–5 cm, more than 4 strictures, or a pre-stricture dilation >5 cm are not optimal for endoscopic management, and surgical intervention is generally preferred [126]. In addition, complex stenoses, such as those associated with fistulas or refractory to previous endoscopic techniques, should be directed towards surgical management. Endoscopic balloon dilation (EBD) is the most used method in current practice for endoscopic therapeutic management, although for deeply ulcerated stenoses it should be considered contraindicated, due to complications [127]. Associated fistulas have classically been regarded as a contraindication to the procedure due to the risk of perforation. Nevertheless, EBD may be performed if the fistulous opening is more than 5 cm from the stricture, allowing adequate luminal drainage [128]. Special attention should be given to strongly angulated strictures with adhesions in CD patients when this procedure is considered, due to the risk of perforation [129]. Any 1 cm increase in stricture length results in an 8% higher risk of surgical intervention [130]. Regarding the technique, a safer approach consists of initiating dilation with a small balloon diameter, such as 12 mm, and gradually increasing it to approximately 15 mm, with the possibility of adjusting the diameter according to the characteristics of the stricture and with the aim of minimizing the risk of perforation. Commonly, the dilation lasts between 30 s and 20 min [129]. Literature data have shown that technical success with EBD can reach up to 89%, and immediate symptom relief has been observed in as many as 81% of patients. However, a significant proportion, around 70%, require repeat procedures [131].

Endoscopic stricturotomy (ES) involves performing a radial incision, including or not circumferential cutting of the fibrotic tissue and optional subsequent placement of clips, and it is used for the treatment of short strictures ≤4 cm (usually involving a single stricture), either as a primary therapeutic option or in refractory strictures. It is considered most suitable for ileocecal, rectal, or pyloric strictures, with limited indications for strictures located in the small intestine [126]. Mohy-Ud-din et al. highlighted the safety and efficacy of the procedure, and previous data have also reported a higher efficacy of ES in patients with CD, with a lower risk of perforation, probably due to the endoscopist’s ability to control the length and depth of the incision, but with a higher hemorrhagic risk compared to EBD [132]. Moreover, ES represents a safe and effective alternative for the management of post-surgical anastomotic strictures in patients with IBD [126,133].

Endoscopic stenting of intestinal strictures has limited data, the procedure being associated with a substantial risk of complications, such as stent migration [132]. A study conducted by Chandan et al. reported that the overall rate of spontaneous distal stent migration was 36.6%, and repeat stenting for symptom management was necessary in 9.6% of patients [131]. Another limitation is the recommendation to remove the metal stent one month after its placement [130].

Endoscopic intralesional injections of steroids or anti-TNF-α agents, including infliximab and adalimumab, require further investigation prior to potential clinical implementation [130]. Table 2 presents the main endoscopic techniques and their clinical relevance for the management of IBD-associated strictures, especially in CD [126,127,128,129,130,131,132,133].

## 5. Fistulas in IBD

Fistula represents pathological communication between two or more epithelial-lined surfaces, and in long-standing IBD, the cumulative incidence of fistulas shows a progressive increase [128]. Fistulas frequently develop in the presence of underlying inflammation. The applicability of this principle is limited in the case of perianal fistulas [134]. Fistula and abscess formation frequently occur above strictures, in an intestinal area under increased pressure, and are characterized by transmural inflammation [117]. The association between fistulas and strictures is not mandatory, although more than 50% of internal fistulas coexist with strictures [128].

### 5.1. Classification and Epidemiology of Fistulas in CD

Fistulas are classified as external and completely internal. External fistulas include perianal and enterocutaneous fistulas, whereas internal fistulas establish communication between different segments of the gastrointestinal tract or between the intestine and adjacent organs, such as the urinary bladder or uterus [135]. Fistula formation between two segments of the gastrointestinal tract is the most frequently encountered type of internal fistula in CD, and symptoms are often closely related to the length and intestinal segment that is bypassed. Thus, fistulas may be asymptomatic if only a short segment of the intestine is bypassed. When a fistula forms between the intestine and adjacent organs, as in enterovesical fistulas, it commonly presents clinically with characteristic symptoms, such as urinary tract infections, pneumaturia, or faecaluria [128]. In a study conducted by Schwarts, the cumulative incidence of fistulas was 26% at 5 years, 33% at 10 years and increased to 50% at 20 years following the diagnosis of CD [136]. In patients with CD affecting the colon and rectum, perianal fistulas are much more common than in those with isolated ileal disease [137].

#### 5.1.1. Pathophysiology

The process of epithelial–mesenchymal transition (EMT) is strongly implicated, as a key mechanism in the development of fibrosis and fistulas in CD [138,139]. The intestinal microbiota may also contribute to fistula formation, as suggested by findings showing increased abundance of *Corynebacterium* and *Achromobacter* in perianal fistula tracts compared with stool samples, although the data remains limited and contradictory [140]. On the other hand, in another study, peptidoglycans (PG) were identified in the fistulous tracts, while the number of bacteria was extremely low. PG potentially inducing a host inflammatory response through the possible secretion of IL-1β, suggesting a possible role of PG in the maintenance of fistulas [137,141].

#### 5.1.2. Perianal Fistulas: Classification and Management

Perianal fistulas may occur prior to the diagnosis of CD and approximately one-third of patients experience this manifestation [142,143]. In some patients, perianal disease may occur as an isolated manifestation, in the absence of any signs of luminal involvement [142]. According to the anatomical relationship of the fistulous tract to the dentate line, perianal fistulas are classified as low or high. Additionally, based on their complexity they may be characterized as simple—typically low-lying, without pain, exhibiting one external opening, or complex—defined by a high location, frequent pain, numerous external openings, and may involve a rectovaginal fistula, anorectal stricture, or active rectal inflammation observed via colonoscopy [135]. The presence of these lesions classifies the disease as an aggressive phenotype, with patients presenting symptoms that include pain in the anal region accompanied by purulent secretion [143]. Up to half of patients with CD may develop fistulas, particularly in the perianal region, which often require surgical intervention despite optimized medical treatment with antibiotics, immunosuppressants, and/or anti-TNF-α therapy [118]. Pelvic MRI with contrast should be used to evaluate perianal disease. Examination under anesthesia performed by an expert surgeon has the highest diagnostic sensitivity and can also facilitate concomitant surgical management. Moreover, it optimizes the performance of MRI and endoscopic ultrasound in identifying fistulas. Low fistulas heal better with fistulotomy, especially in the absence of proctitis, whereas high fistulas or those associated with proctitis have poorer healing and a higher risk of incontinence [144]. Recurrent fistulas occur in about one-third of patients despite modern therapeutic and surgical advances [136,140]. Moreover, there is evidence of malignant transformation of perianal fistulas in patients with CD [145,146].

### 5.2. Fistulas in UC

Patients with severe forms of UC who do not respond to medical therapy may undergo proctocolectomy with ileal pouch–anal anastomosis (IPAA), and fistulas may occur between the pouch and the adjacent organs, including genitourinary tract. Lo et al. reported an overall fistula prevalence of 6% among this category of patients [147]. Pelvic sepsis or CD can cause fistulas; traditionally, those that appear early after surgery are classified in the first category, while those that appear later fall into the second category. Recent data suggest that pelvic sepsis can lead to fistulas even several years after surgery [148]. One of the fistulas that may occur is pouch–vaginal fistula, with important consequences [149]. This may be classified as low or high, the former being more common. Low pouch–vaginal fistulas often result from dehiscence of IPAA or from accidental involvement of the vaginal wall intraoperatively. Pouch–vaginal fistula represents a significant complication, and one of its most profound consequences is pouch failure [150]. Pouch failure leads to an abdominoperineal pouch revision or a permanent stoma [148]. Up to 15% of patients may experience failure of primary IPAA, while those undergoing secondary pouch intervention have a higher rate of pouch failure compared to that observed after primary surgery [151]. PSC is associated with poorer pouch function and increased rates of pouchitis [148].

### 5.3. The Role of Endoscopy in IBD Fistulas

Endoscopy may contribute as a therapeutic tool in management of fistulas in CD. There is no standardized endoscopic treatment for these patients. Over time, various endoscopic techniques have been attempted for fistula closure, such as the placement of hemostatic clips, the use of endoscopic suturing devices, or of self-expandable metal stents with the aim of closing the fistulous tract. Some fistulas can be managed by endoscopic fistulotomy, with subsequent placement of endoclips to prevent reconnection of the fistulous tract. The Over-The-Scope Clip system is being used increasingly widely due to its greater tissue-grasping capacity, but data regarding its efficacy in this category of patients are still limited. According to Kochhar and Shen, endoscopic clipping remains a rescue option, considered when patients are not eligible for fistulotomy [134]. Moreover, abscesses associated with perianal fistulas can be drained endoscopically using a needle knife, while some intra-abdominal abscesses can be drained endoscopically using a pigtail stent, with the possibility of endoscopic ultrasound (EUS) guidance [126,152]. For successful endoscopic fistulotomy, fistulas should be superficial, have a single tract, be short (<3–4 cm), and be located in the distal intestine or perianal region [134,152]. The use of adipose tissue-derived stem cells, autologous or allogeneic, for the treatment of perianal fistulas is presently a major subject of scientific interest [153]. A meta-analysis by Aldakhil et al. reported an average closure rate of 62.5% for complex perianal fistulas treated with adipose-derived stem cells [154]. The main endoscopic approaches to fistulas in CD such as techniques, indications, and limitations are summarized in Table 3 [126,134,152,153,154].

## 6. Persistent Effects of Chronic Inflammation on Anorectal Sensorimotor Function in IBD Patients in Remission

Rectal inflammation underlies the development of numerous symptoms in UC. Reduced rectal compliance has been observed in patients with UC and may be associated with functional symptoms, even when endoscopic findings no longer show active inflammation [7]. These functional symptoms, such as increased stool frequency, urgency, and fecal incontinence, are like those seen in active disease, creating diagnostic difficulties [12]. The advances made in achieving mucosal healing in patients with IBD contrast with the persistence of defecatory symptoms, which may be partly attributable to inflammatory neuromuscular sequelae (but are not limited to them), affecting the sensorimotor function of the anorectum [155]. Intestinal motility results from the coordinated action of myenteric neurons on the smooth muscle and the activity of interstitial cells of Cajal. This enteric nervous system, although connected to the central nervous system, is capable of exerting autonomous control over intestinal functions [156]. Oxidative stress associated with inflammation contributes to the damage of colonic smooth muscle DNA, which compromises muscle function and may persist even after the resolution of acute inflammation. Additionally, excessive NO production is implicated in motility dysfunction in UC [7], along with increased levels of IL-1β and the increased expression of COX-2 in the myenteric neurons [157]. COX-2 mRNA was also detected by in situ hybridization analysis in neuronal cells of the myenteric plexus, smooth muscle cells, and inflammatory cells in the lamina propria of IBD samples [59]. In patients with UC, the inflammatory process has been associated with a reduction in the number of enteric neurons in the myenteric plexus. This reduction is irreversible even after resolution of inflammation, with neutrophil infiltration in the enteric ganglia proposed as one of the mechanisms leading to neuronal death [156]. Some authors describe changes at the neuronal level such as hypertrophy, hyperplasia, and axonal damage of nerve fibers, as well as similar changes affecting neuronal cell bodies. Although changes in the enteric nervous system have traditionally been regarded as secondary to the inflammatory process, the presence of lesions in areas without visible structural changes suggests that these abnormalities may precede the onset of inflammation [158]. In line with this, ganglioneuritis has been observed even in colonic segments without active inflammation in patients with UC [7]. Villanacci et al. observed in patients with CD a reduction in enteric glial cells in the submucosal plexus, including in areas unaffected by the disease, compared with the control group. In addition, myenteric plexitis was observed in approximately three quarters of patients with CD and in about half of patients with UC, in the context of inflammation limited to mucosa. Plexitis may be associated with disease progression [158]. Studies on the morphological changes in the enteric nervous system are, however, heterogeneous [7,158]. Moreover, a variety of excitatory and inhibitory neurotransmitters are released by motor neurons that innervate the circular and longitudinal muscle layers. Contraction is largely mediated by acetylcholine and substance P, while relaxation is mediated by VIP, NO and purines [156]. Altered neurotransmitter profiles have also been reported in patients with IBD [158,159]. Therefore, inflammation-mediated enteric neuroplasticity underlies impaired colonic motility in IBD, both during active disease and in remission [160].

The motility of the gastrointestinal tract, from the pharynx [161,162] to the intestinal segments, has been the subject of extensive and detailed studies over time using manometry [163]. Colonic motility has been previously investigated in various studies, using different methodologies, some of which identified decreased or absent colonic motility in patients with UC as well as the presence of paradoxical motility [157]. High-resolution anorectal manometry is the one of the most important investigations for evaluating anorectal sensorimotor function [14]. It can analyze anal sphincter strength, rectal sensitivity, rectal compliance, anorectal coordination during simulated defecation, the rectoanal inhibitory reflex, and overall evacuatory function, including balloon expulsion test [12].

Underdiagnosis of these sensorimotor disturbances may result in unnecessary investigations, unwarranted escalation of therapy and impairment of patients’ quality of life [12].

Table 4 highlights a synthesis of the major long-term complications of IBD (dysplasia and CRC, stenosis, fistulas, anorectal sensorimotor dysfunction), integrating their pathophysiological mechanisms driven by chronic inflammation, together with key risk factors and clinical and therapeutic implication [7,9,12,20,26,27,49,116,117,118,119,120,125,128,135,138,139,147,148,149,150,157].

## 7. Conclusions

Chronic, cumulative inflammation in IBD extends beyond mucosal involvement and has major clinical consequences. It promotes colorectal carcinogenesis, increasing the risk of CRC, and drives progressive transmural remodeling, resulting in fibrosis, strictures, fistulas, and anorectal sensorimotor dysfunction. These features reflect persistent, inadequately controlled inflammation and represent key determinants of structural disease progression and impaired quality of life. Understanding the mechanisms sustaining chronic inflammation is therefore essential, not only for elucidating disease pathogenesis, but also for identifying therapeutic targets capable of modifying disease course and preventing both structural and neoplastic complications, particularly in the era of mucosal healing. In recent years, histological healing has emerged as a potential therapeutic endpoint beyond endoscopic remission. In this context, future directions include the development of individualized surveillance strategies based on cumulative inflammatory burden and the integration of histological healing into risk stratification models. Endoscopy remains central for diagnosis, disease monitoring, and dysplasia surveillance in IBD, contributing to CRC prevention through structured surveillance programs. Its effectiveness depends on adherence to follow-up protocols and patient compliance. In expert centers, advanced endoscopic techniques may offer minimally invasive alternatives for selected strictures and fistulizing disease, reducing the need for surgery.

Finally, the assessment of sensorimotor dysfunction in IBD remains complex and insufficiently standardized, highlighting the need for validated protocols to support the integration of functional assessment into routine clinical practice. Patients with IBD should be evaluated using a holistic approach, as persistent functional disorders during remission and manometric abnormalities further support this strategy.

## Figures and Tables

**Table 1:** Endoscopic surveillance techniques for the detection of IBD-associated neoplasia and their clinical applicability.

Method	Clinical Application
SD-WLE	Old method Replaced by modern techniques
DCE	Preferred method in current clinical practice Delineates lesion margins Increases neoplasia detection
HD-WLE	Recommended Improves detection accuracy Performance close to DCE in some studies
HD-WLE + DCE	Highest diagnostic performance
VCE (NBI)	Alternative to DCE Similar efficacy in neoplasia detection
CLE	Real-time in vivo histologic evaluation Limited clinical availability
EC	Very detailed assessment of crypt and nuclear morphology Limited clinical availability
ME	Emerging technique Fluorescently labeled antibodies or peptides targeting dysplasia-associated proteins

Abbreviations: SD-WLE: standard-definition white-light endoscopy, DCE: dye-based chromoendoscopy, HD-WLE: high-definition white-light endoscopy, HD-WLE-DCE: high-definition white-light endoscopy with dye-based chromoendoscopy, VCE (NBI): virtual chromoendoscopy (with narrow-band imaging), CLE: confocal laser endomicroscopy, EC: endocytoscopy, ME: molecular endoscopy.

**Table 2:** Endoscopic management of strictures in IBD.

Endoscopic Technique	Indications	Limitations	Advantages	Complications
EBD	CD: Fibrotic or mixed strictures amenable to endoscopic therapy (length ≤4–5 cm, ≤4 strictures, pre-stenotic dilation ≤5 cm) In IBD, particularly UC: biopsy required for CRC differential diagnosis	Deeply ulcerated strictures Complex strictures associated with fistulas Strictures requiring surgical management: length >4–5 cm, >4 strictures, or pre-stenotic dilation >5 cm	Most used technique Effective and safe in selected cases	Risk of perforation (higher in angulated strictures in CD)—stepwise balloon dilation starting at ~12 mm and gradually increasing to ~15 mm, with adjustment based on stricture characteristics Need for repeat procedures
ES	CD: short strictures (≤4 cm) and usually involving a single stricture Refractory strictures Most suitable ileocecal, rectal, or pyloric strictures	Limited use in small bowel strictures	Higher efficacy and lower risk of perforation compared with EBD Safe and effective alternative for postoperative anastomotic strictures	Higher bleeding risk compared with EBD
Endoscopic stenting	Selected cases of intestinal strictures	Limited evidence Stent removal recommended after 1 month	May relieve obstructive symptoms	Frequent stent migration
Intralesional injections (steroids, anti-TNF-α agents)	Subject of ongoing research	Insufficient evidence	Potential therapeutic option	Further studies required before routine clinical use

Abbreviations: EBD: Endoscopic Balloon Dilation, CD: Crohn’s Disease, IBD: Inflammatory Bowel Disease, UC: Ulcerative Colitis, CRC: Colorectal Cancer, ES: Endoscopic Stricturotomy, anti-TNF-α: anti-Tumor Necrosis Factor alpha.

**Table 3:** The main endoscopic approaches to fistulas in CD.

Category	Endoscopic Technique	Clinical Role	Key Limitations
Fistula closure techniques	Endoscopic clipping (hemostatic clips), OTSC, system, endoscopic suturing, self-expandable metal stents	Closure of fistulous tract	Limited evidence—mainly rescue or adjunctive use
Direct fistula treatment	Endoscopic fistulotomy	Endoscopic management with clip placement to prevent recurrence in selected cases (limited evidence)	Suitable only for superficial, single, short fistulas (<3–4 cm)
Abscess management	Endoscopic abscess drainage (needle-knife, pigtail stent ± EUS guidance), selected according to abscess location	Drainage of perianal or intra-abdominal abscesses	Requires expertise
Regenerative therapy	Adipose-derived stem cell therapy	Treatment of complex perianal fistulas	Investigational therapy

Abbreviations: OTSC: The Over-The-Scope Clip system, EUS: endoscopic ultrasound.

**Table 4:** Overview of the major long-term complications of IBD.

Complication	Pathogenesis and Link to Chronic Inflammation	Risk Factors	Clinical and Therapeutic Relevance
Colonic dysplasia and CRC	Chronic inflammation drives oxidative stress and DNA damage → genomic and epigenetic instability	persistent histologic inflammation, long disease duration, extensive colitis, PSC, history of dysplasia (especially multifocal or high-grade), family history of CRC	premalignant lesion with variable risk of progression; requires intensified and personalized surveillance and individualized management, including consideration of colectomy in high-risk cases
Stenosis in CD	Result of a combination of inflammatory processes and fibrosis, which develops following excessive extracellular matrix accumulation driven by long-term inflammation	age under 40 at diagnosis, complete colonic involvement, ileocecal disease location, long disease course, perianal disease at diagnosis	disease progression associated with cumulative structural damage; increased risk of complications and surgery over time need for imaging differentiation between inflammatory and fibrotic strictures
Stenosis in UC	Luminal narrowing due to chronic inflammation and structural remodeling, causing obstruction with proximal bowel dilation	long disease duration, PSC, presence of histological inflammation	associated with lower remission rates, higher complication rates, and increased need for surgical treatment colorectal strictures require careful evaluation due to a high association with dysplasia and CRC
Fistulas in CD	Transmural inflammation drives fistula formation epithelial–mesenchymal transition contributes to fibrosis and fistulization	long disease duration, colonic and rectal involvement (increased perianal fistulas), presence of strictures (often coexist in >50% of cases)	progressive complication with increasing cumulative incidence over time fistulas may be asymptomatic or symptomatic depending on location; simple and complex forms with different clinical severity and management approaches high recurrence rate (~1/3); often requiring medical and/or surgical treatment
Fistulas in UC	Usually occurs post-surgically following proctocolectomy with IPAA in patients with severe UC requiring surgical treatment due to lack of adequate response to medical therapy	pelvic sepsis (postoperative complication), underlying or misdiagnosed CD PSC is associated with poorer pouch function	occur in approximately 6% of patients following IPAA and include pouch–vaginal fistulas, associated with pouch failure (may require surgical revision or permanent stoma formation)
Anorectal sensorimotor dysfunction	Chronic inflammation induces oxidative stress and enteric neuronal injury and loss Altered neurotransmitter balance induces impaired motility and anorectal dysfunction persisting despite mucosal healing	history of UC or CD with rectal involvement, persistent or prior intestinal inflammation, inflammatory enteric neuromuscular changes (including plexitis and ganglioneuritis)	persistent anorectal symptoms despite endoscopic remission, including urgency, increased stool frequency, and fecal incontinence clinical overlap with active disease may lead to underdiagnosis, unnecessary investigations, and overtreatment anorectal manometry allows assessment of sphincter function, rectal sensitivity, and defecatory disorders

Abbreviations: CRC: Colorectal Cancer, PSC: Primary Sclerosing Cholangitis, CD: Crohn’s Disease, UC: Ulcerative Colitis, IPAA: Ileal Pouch–Anal Anastomosis.

## Data Availability

No new data were created or analyzed in this study. Data sharing is not applicable to this article.
